# Supplementary material for: Content-rich biological network constructed by mining PubMed abstracts
Source: BMC Bioinformatics. 2004 Oct 8;5:147. doi: 10.1186/1471-2105-5-147 (PMC528731; doi:10.1186/1471-2105-5-147)
Supplement: Additional File 5 — The original Chilibot query results of the term "long-term potentiation (LTP)" and 22 other terms, limiting the latest references analyzed to the years 1990, 1995, 2000, and 2004. [file 1471-2105-5-147-S5.bz2 › chilibotAdditionalFile5/ltp2004/html/LTP_PKA.html]

 


 **LTP** and **PKA** 
  
Found 96 abstracts in PubMed,  **30 abstracts were retrieved and analyzed**.  


---

 Search Google  |
 PDF files only 
|  EDU domain only 

---

**Interactive relationship** (e.g. stimulation, inhibition, etc)

- Our data show that genetic inhibition of  **PKA**  impairs L  **LTP**  by reducing  **PKA**  mediated suppression of PP1 2A.  Ref: 12453050 Eur J Neurosci, 2002
- One way in which  **PKA**  may critically modulate L  **LTP**  is by relieving an inhibitory constraint imposed by protein phosphatases.  Ref: 12453050 Eur J Neurosci, 2002
- We have shown that  **PKA**  inhibitors have a major effect on both  **LTP**  and LTD in the visual cortical slices.  Ref: 12853373 Cereb Cortex, 2003
- However, inhibition of CaMKII together with inhibition of either  **PKA**  or PKC fully blocked the induction of  **LTP** .  Ref: 14529720 Neuropharmacology, 2003
- This heterosynaptically induced L  **LTP**  requires  **PKA**  and protein synthesis.  Ref: 14711997 Proc Natl Acad Sci U S A, 2004
- Presynaptic activation of protein kinase A  **PKA**  induces  **LTP**  in cerebellar parallel fiber synapses.  Ref: 14532002 Cell, 2003
- Activation of apostsynaptic  **PKA**  is necessary for the expression of  **LTP**  in CA1 pyramidal neurons and is sufficient for initiating persistent synaptic facilitation.  Ref: 12598602 J Neurosci, 2003
- The late phase of  **LTP**  L  **LTP**  requires activation of cAMP dependent protein kinase  **PKA**  for its full expression.  Ref: 12453050 Eur J Neurosci, 2002
- These genetic and pharmacological experiments show that  **LTP**  induced by these compressed patterns of stimulation does not require  **PKA**  activation.  Ref: 12699336 Hippocampus, 2003
- Paired pulse facilitation was unchanged during most of the duration of  **LTP** , suggesting that postsynaptic mechanisms, including  **PKA**  activation, are involved in the expression of  **LTP**  induced by multitrain stimulation.  Ref: 12598602 J Neurosci, 2003
- The  **PKA**  activator, 8 chloroadenosine monophosphorothioate, Sp isomer Sp 8 Cl cAMPS, by itself induces a slowly rising form of  **LTP** , which is occluded by theta burst stimulation TBS induced  **LTP** .  Ref: 12853373 Cereb Cortex, 2003
- Our findings suggest that  **PKA**  mediated phosphorylation of the active zone protein RIM1alpha at a single N terminal site induces presynaptic  **LTP** .  Ref: 14532002 Cell, 2003
- These experiments reveal, unexpectedly, the existence of two parallel kinase pathways, one involving CaMKII and the other  **PKA**  and PKC, either of which can fully support the induction of  **LTP** , at this stage of development.  Ref: 14529720 Neuropharmacology, 2003
- Long term potentiation  [ **LTP** ]   **LTP**  and long term depression LTD at thalamocortical synapses require postsynaptic protein kinase A  **PKA**  activity and are difficult to induce in barrelless mice, probably due to an inability to properly regulate synaptic AMPAR trafficking.  Ref: 12897788 Nat Neurosci, 2003
- These results suggest that activation of CaMKII,  **PKA**  and PKC may be crucial for the induction and the early phase but not for the late phase maintenance of the spinal  **LTP** .  Ref: 14586032 J NeurophysiolJ Neurophysiol, 2003
- Multiple trains of high frequency synaptic stimulation evoke long term potentiation  [ **LTP** ]   **LTP**  of synaptic transmission in hippocampal area CA1, which has been correlated with hippocampal long term memory and requires the activation of cAMP dependent protein kinase  **PKA** .  Ref: 12598602 J Neurosci, 2003
- Phosphorylation of RIM1alpha by  **PKA**  triggers presynaptic long term potentiation  [ **LTP** ]  at cerebellar parallel fiber synapses.  Ref: 14532002 Cell, 2003
- Considerable evidences have shown that calcium calmodulin dependent protein kinase II CaMKII, protein kinase A  **PKA**  and protein kinase C PKC are important for  **LTP**  in hippocampus.  Ref: 14586032 J NeurophysiolJ Neurophysiol, 2003

**Parallel relationship** (e.g. studied together, co-existance, homology, etc.)

- These results suggest that NO, cGMP, and PKG cause release of calcium from ryanodine sensitive stores, which in turn causes phosphorylation of CREB in parallel with  **PKA**  during the induction of L  **LTP** .  Ref: 12205148 J Neurophysiol, 2002
- In particular, phosphorylation of GluR1, an AMPAR subunit, by  **PKA**  at serine 845 S845 increases peak open channel probability and is permissive for both the synaptic expression of the receptor and NMDA receptor NMDAR dependent long term potentiation  [ **LTP** ]   **LTP** .  Ref: 12843287 J Neurosci, 2003
- In contrast,  **LTP**  induced by temporally compressed tetraburst stimulation was normal in slices from R AB mutants, and its long term maintenance was not significantly affected by bath application of KT 5720, an inhibitor of catalytic subunits of  **PKA** .  Ref: 12699336 Hippocampus, 2003
- To assess whether postsynaptic  **PKA**  is necessary for the expression of  **LTP** , we made prolonged whole cell voltage clamp recordings from CA1 pyramidal neurons in mouse hippocampal slices during postsynaptic infusion of cell impermeant modulators of  **PKA** .  Ref: 12598602 J Neurosci, 2003
- **PKA**  dependent  **LTP**  is selectively recruited by temporally spaced, multiburst synaptic stimulation.  Ref: 12699336 Hippocampus, 2003
- We previously found that the nitric oxide NO cGMP cGMP dependent protein kinase PKG signaling pathway acts in parallel with the cAMP cAMP dependent protein kinase  **PKA**  pathway to produce protein and RNA synthesis dependent late phase long term potentiation  [ **LTP** ]  L  **LTP**  and cAMP response element binding protein CREB phosphorylation in the CA1 region of mouse hippocampus.  Ref: 12205148 J Neurophysiol, 2002
- Previous studies documented an integral role for the cAMP  **PKA**  system in late  **LTP**  and recently, inhibition of cAMP degradation facilitates  **LTP**  and ameliorates mnemonic deficits.  Ref: 12617967 Neuroscience, 2003
- Using mutant  **PKA**  mice R AB transgenic mice that have genetically reduced hippocampal  **PKA**  activity, we show that deficient L  **LTP**  in area CA1 of mutant hippocampal slices is rescued by acute application of two inhibitors of protein phosphatase 1 and protein phosphatase 2A PP1 2A okadaic acid and calyculin A.  Ref: 12453050 Eur J Neurosci, 2002
- Genetic and electrophysiological experiments have defined an important role for cAMP dependent protein kinase  **PKA**  in certain forms of long term potentiation  [ **LTP** ]   **LTP** .  Ref: 12699336 Hippocampus, 2003
- The Roles of CaMKII,  **PKA**  and PKC in the Induction and Maintenance of  **LTP**  of C Fiber Evoked Field Potentials in Rat Spinal Dorsal Horn.  Ref: 14586032 J NeurophysiolJ Neurophysiol, 2003
- Previous reports show that bath application of the adenosine 3 5 cyclic monophosphate cAMP analog, Sp cAMPS, induces a protein kinase A  **PKA**  dependent and protein synthesis dependent long term potentiation  [ **LTP** ]   **LTP**  at hippocampal CA3 CA1 synapses.  Ref: 12710011 Synapse, 2003
- Thus, altering the temporal spacing of synaptic stimulation per se critically modulates the  **PKA**  dependence of hippocampal  **LTP** .  Ref: 12699336 Hippocampus, 2003
- The facilitation of hippocampus based, long lasting synaptic plasticity, which is frequently investigated in model systems such as long term potentiation  [ **LTP** ]   **LTP**  and in learning paradigms such as the Morris water maze, is associated with several cellular key events calcium influx through the N methyl D aspartate NMDA receptor, generation of cyclic AMP cAMP and activation of protein kinase A  **PKA** , phosphorylation of mitogen associated protein kinase MAPK and cAMP response element binding protein CREB, and subsequent transcription of plasticity associated genes.  Ref: 12668903 Mol Neurobiol, 2003
- Amyloid beta peptide inhibition of the  **PKA**  CREB pathway and long term potentiation  [ **LTP** ]  reversibility by drugs that enhance cAMP signaling.  Ref: 12244210 Proc Natl Acad Sci U S A, 2002
- These results support the point that the  **PKA**  signaling pathway is crucial for neuronal plasticity in visual cortex, and the dissociation of the role of  **PKA**  and PKG in long term synaptic plasticity in the visual cortex suggests that  **LTP**  alone is not sufficient to support ocular dominance plasticity, or LTD plays a more fundamental role than  **LTP**  in ocular dominance plasticity.  Ref: 12853373 Cereb Cortex, 2003
- These observations suggest that Alphabeta acts directly on the pathways involved in the formation of late  **LTP**  and agents that enhance the cAMP  **PKA**  CREB signaling pathway have potential for the treatment of AD.  Ref: 12244210 Proc Natl Acad Sci U S A, 2002
- In the present study, we have used  **PKA**  mutant mice R AB transgenic mice, which have reduced  **PKA**  activity in neurons within the hippocampus, to explore the role of temporal spacing of synaptic stimulation in regulating the  **PKA**  dependence of  **LTP** .  Ref: 12699336 Hippocampus, 2003
- We have now tested the role of  **PKA**  and PKG in long term potentiation  [ **LTP** ]   **LTP**  and long term depression LTD.  Ref: 12853373 Cereb Cortex, 2003
- We report here that, unlike  **LTP**  in the more mature CA1 region of the hippocampus,  **LTP**  in neonatal rodent hippocampus < ; 9 postnatal days, < ; P9 requires cyclic AMP dependent protein kinase A  **PKA**  but not calcium calmodulin dependent protein kinase II CaMKII.  Ref: 12469130 Nat Neurosci, 2003
- However, the characteristics of stimulation that are critical for regulating the  **PKA**  dependence of  **LTP**  have not been clearly defined.  Ref: 12699336 Hippocampus, 2003
- In Str slices treated with the PKC and or  **PKA** , the same stimulation induced long term potentiation  [ **LTP** ]  of field potentials similar to those observed in slices obtained from MW rats.  Ref: 12012161 Exp Brain Res, 2002
- In present work the roles of these three protein kinases in the induction and maintenance of  **LTP**  of C fiber evoked field potentials were evaluated by application of specific inhibitors of CaMKII KN 93 and AIP,  **PKA**  Rp CPT cAMPS and PKC chelerythrine and Go 6983 at the recording segments before and after  **LTP**  induction in Urethane anesthetized Sprague Dawley rats.  Ref: 14586032 J NeurophysiolJ Neurophysiol, 2002
